# Regional distribution and severity of arterial calcification in patients with chronic kidney disease stages 1–5: a cross-sectional study of the Copenhagen chronic kidney disease cohort

**DOI:** 10.1186/s12882-020-02192-y

**Published:** 2020-12-09

**Authors:** Ida Maria Hjelm Sørensen, Sasha Asbøll Kepler Saurbrey, Henrik Øder Hjortkjær, Philip Brainin, Nicholas Carlson, Ellen Linnea Freese Ballegaard, Anne-Lise Kamper, Christina Christoffersen, Bo Feldt-Rasmussen, Klaus Fuglsang Kofoed, Susanne Bro

**Affiliations:** 1 Department of Nephrology, Rigshospitalet University Hospital, Blegdamsvej 9, DK-2100 Copenhagen, Denmark; 2 Department of Cardiology, Rigshospitalet University Hospital, Blegdamsvej 9, DK-2100 Copenhagen, Denmark; 3 Department of Cardiology, Herlev and Gentofte University Hospital, Niels Andersens Vej 65, Post 835, DK-2900 Copenhagen, Denmark; 4 Department of Clinical Biochemistry, Rigshospitalet University Hospital, Blegdamsvej 9, DK-2100 Copenhagen, Denmark; 5 Department of Biomedical Sciences, University of Copenhagen, Blegdamsvej 3B, DK-2200 Copenhagen, Denmark

**Keywords:** Chronic kidney disease, Cardiovascular disease, Vascular calcification, Arterial calcification, Calcium score, Carotid arteries, Coronary arteries, Thoracic aorta, Abdominal aorta, Iliac arteries

## Abstract

**Background:**

Patients with chronic kidney disease (CKD) and arterial calcification are considered at increased risk of adverse cardiovascular outcomes. However, the optimal site for measurement of arterial calcification has not been determined. The primary aim of this study was to examine the pattern of arterial calcification in different stages of CKD.

**Methods:**

This was an observational, cross-sectional study that included 580 individuals with CKD stages 1–5 (no dialysis) from the Copenhagen CKD Cohort. Calcification of the carotid, coronary and iliac arteries, thoracic and abdominal aorta was assessed using non-contrast multidetector computed tomography scans and quantified according to the Agatston method. Based on the distribution of Agatston scores in the selected arterial region, the subjects were divided into calcium score categories of 0 (no calcification), 1–100, 101–400 and > 400.

**Results:**

Participants with CKD stages 3–5 had the highest prevalence of calcification and the highest frequency of calcium scores > 400 in all arterial sites. Calcification in at least one arterial site was present in > 90% of patients with CKD stage 3. In all five CKD stages prevalence of calcification was greatest in both the thoracic and abdominal aorta, and in the iliac arteries. These arterial sites also showed the highest calcium scores. High calcium scores (> 400) in all five arterial regions were independently associated with prevalent cardiovascular disease. In multivariable analyses, after adjusting for cardiovascular risk factors, declining creatinine clearance was associated with increasing calcification of the coronary arteries (*p* = 0.012) and the thoracic aorta (*p* = 0.037) only.

**Conclusions:**

Arterial calcification is highly prevalent throughout all five CKD stages and is most prominent in both the thoracic and abdominal aorta, and in the iliac arteries. Follow-up studies are needed to explore the potential of extracardiac calcification sites in prediction of cardiovascular events in the CKD population.

## Background

Risk of cardiovascular disease is increased in patients with chronic kidney disease (CKD) [1, 2]. Likewise, the prevalence of CKD is increasing [3] and with it the need for cardiovascular risk prediction and intervention.

Despite the high prevalence of traditional risk factors, cardiovascular risk prediction developed in general populations underestimates the risk of cardiovascular disease in CKD populations [4]. An alternative approach for predicting symptomatic cardiovascular disease is by assessment of asymptomatic cardiovascular disease, evaluating the calcium score of the arteries using non-contrast multidetector computed tomography (MDCT).

The coronary artery calcium score (CACS) correlates with the total plaque burden of the coronary arteries [5] and predicts future cardiovascular events for the individual person much better than risk factor-based scoring systems in the general population [6, 7]. Also in CKD, CACS provides additional value beyond existing clinical risk factor scoring systems [8–11] .

Arterial calcification is characterized by two pathologically different processes, indistinguishable by CT scan. Intimal calcification is predominant in the general population, whereas medial calcification is common in CKD, old age and diabetes [12]. On this basis, we hypothesized that CACS or calcification in other arterial sites might predict other cardiovascular events in the CKD population as compared with the general population. Moreover, since intimal and medial calcification seem to be independent processes, it is questionable whether CACS (or CACS alone) is the strongest predictor of future cardiovascular disease in CKD.

The Copenhagen (CPH) CKD Cohort Study is the first to examine the distribution and severity of arterial calcification using non-contrast MDCT scanning of five major arterial sites in patients with CKD stages 1–5. Our primary aim was to examine the pattern of arterial calcification in different stages of CKD. Our secondary aim was to evaluate the relation between degree of arterial calcification, cardiovascular risk factors, including decreased kidney function and prevalent cardiovascular disease.

## Methods

### Study population

The present study was a cross-sectional analysis of baseline data from the CPH CKD Cohort Study, which is a single centre, prospective, observational study examining risk factors for progression of cardiovascular disease and advanced imaging methods for early detection of cardiovascular disease in a cohort of individuals aged 30–75 years with any diagnosis of CKD stages 1–5. This paper describes a novel cohort inspired by and based upon experience from a smaller, less detailed cohort [13].

In accordance with the 2012 Kidney Disease Improving Global Outcomes (KDIGO) Guidelines [14], CKD was defined as either kidney damage or estimated glomerular filtration rate (eGFR) < 60 mL/min per 1.73 m^2^ of body surface for ≥3 months. We defined albuminuria or proteinuria as urine albumin/creatinine ratio ≥ 30 × 10^− 3^ or 24-h urine protein ≥0.1 g. In agreement with KDIGO [14] the CKD stages were as follows, CKD stage 1: kidney damage with GFR > 90 (mL/min/1.73m^2^), CKD stage 2: kidney damage with GFR 60–89, CKD stage 3: GFR 30–59, CKD stage 4: GFR 15–29, and CKD stage 5 ND: GFR < 15, no dialysis. eGFR to define eligibility was based on a measured plasma creatinine value and the CKD-EPI_krea_ formula [15].

From October 2015 to June 2017, participants were consecutively recruited from the nephrology outpatient clinic at Rigshospitalet, Copenhagen University Hospital, Denmark. Patients with previous renal transplantation with a functioning graft, active malignancy, pregnancy, and patients with intellectual disability, dementia or psychosis were excluded. Information on baseline demographic characteristics, medical history, medications, side-effects and lifestyle factors were retrieved from patient interviews and electronic medical records. The participants underwent a clinical examination which included measurement of standardized blood pressure, height, weight and mid-abdominal waist circumference. A fasting blood sample and a 24-h urine sample were obtained. Plasma analytes were assessed as described [13].

Cardiovascular disease was defined as a composite of prevalent coronary artery disease, a history of previous cerebrovascular infarction, carotid endarterectomy or stenting and/or peripheral artery disease. Detailed definitions of coronary artery disease and peripheral arterial disease have been previously described [13]. Some participants had more than one cardiovascular diagnosis.

Hypertension was defined as systolic blood pressure > 140 mmHg and/or diastolic blood pressure > 90 mmHg or use of oral antihypertensive treatment. Hypercholesterolemia was defined as low-density lipoprotein (LDL) cholesterol > 3 mmol/l or treatment with cholesterol-lowering medication. Physical activity was categorized as described by Schnohr et al. [16].

The study followed the principles of the Declaration of Helsinki and was approved by the Regional Scientific Ethical Committee (H-3-2011-069) and the Danish Data Protection Agency. All participants signed a written informed consent prior to inclusion.

### MDCT scan

Participants were examined with a 320-detector CT scanner (Aquillon One, Toshiba medical Systems, Japan) using electrocardiography-gated scanning, radiation exposure was 1–10 millisievert. Individuals with a heart rate > 65 bpm were treated with oral betablocker (metoprolol 25–150 mg) or were administered oral ivabradine if betablocker was contraindicated. Non-contrast recordings using 3 mm tomography slices were acquired for assessment of arterial calcification.

### Arterial calcification

Calcification score was assessed according to the Agatston method, where calcium deposits in the arterial wall are identified as a dense area exceeding a threshold of 130 Hounsfield units [17].

*Carotid and coronary arteries.* Calcification scoring of the carotid and coronary arteries was performed as previously described [17–19]. *Thoracic aorta.* The total thoracic aorta calcium score was determined by summing individual lesion scores from each of the three anatomic sites (ascending aorta, aortic arch, descending aorta) and was defined as the segment from the aortic root to the diaphragm. *Abdominal aorta.* The abdominal aorta was defined as the segment from the diaphragm to the iliac bifurcation. *Iliac arteries.* The common iliac arteries were measured from their origin of the abdominal aorta down to their bifurcation into the external and internal iliac arteries. The iliac external arteries were then followed as far as the CT scan permitted. Data from the right and left sides were combined to give the extent of the total iliac calcium score.

Calcified areas were manually registered for each artery and automatically computed using the dedicated software (Vitrea, Vital Images Inc., USA). All lesions in a given vascular bed were added to calculate the total Agatston score. Presence of calcification was defined as an Agatston score > 0. Based on the distribution of Agatston scores in the selected arterial region, the participants were divided into calcium score categories of 0 (no calcification), 1–100, 101–400 and > 400 [20].

Images of inadequate quality were excluded. Inter- and intraobserver variability of each arterial segment was assessed in 50 randomly selected participants. Intraobserver coefficients of variation (CV) varied between 7.0 and 10.3%, interobserver CVs between 7.1 and 12.7%.

### Statistical analyses

Statistical analyses were performed using SPSS version 22 (IBM SPSS Statistics), STATA version 14.1 (StataCorp LP) and R version 3.2.1 (R Foundation for Statistical Computing). A two-sided *p*-value of < 0.05 was deemed statistically significant. Categorical variables are presented as n (%) and compared using the χ^2^-test. Continuous normal distributed variables are presented as the mean (standard error of the mean [SEM]) and compared using one-way analysis of variance (ANOVA). Continuous non-normally distributed variables are presented as the median (interquartile range [IQR]) and compared using the Mann-Whitney U or Kruskal-Wallis test.

Because calcium scores from all arterial sites represented highly dispersed count data, we applied negative binomial regression (NBR) models to assess the relative change in calcium score with 95% confidence intervals (CI) [21]. Unadjusted NBR models were used to assess the association between change in arterial calcium score and cardiovascular risk factors: age, sex, hypertension, diabetes, smoking, use of cholesterol lowering medication, warfarin treatment and creatinine clearance. Creatinine clearance was preferred to eGFR as a measure of kidney function, since the first mentioned is a measured value and the latter only an estimate with no exact value when eGFR > 90 mL/min/1.73m^2^. In addition, we conducted analyses which were mutually adjusted for the above-mentioned risk factors. Cubic spline models based on NBR were used to illustrate the relation between creatinine clearance and arterial calcium score. Number of knots were determined according to the lowest Akaike information criterion. Adjusted multivariable logistic regression models were used to investigate the association between arterial calcification (divided into calcium score categories) and the presence of cardiovascular disease. The rationale behind the covariates included in the fully adjusted models was based on their clinical relevance as risk factors of cardiovascular disease. We reported the results from the regression analyses as odds ratios (ORs) with 95% CIs. Due to nonlinearity of the continuous variables (age, creatinine clearance), these were splined applying restricted cubic spline (5 knots) [22]. Intra- and interobserver variability of calcium scoring were evaluated using CV and Bland-Altman analyses.

## Results

### Characteristics of the study cohort

Among the 741 participants enrolled in the CPH CKD Cohort, a total of 580 accepted to undergo non-contrast MDCT scanning. The 161 patients who declined did not differ significantly from the patients accepting in terms of age, sex, prevalence of diabetes, hypertension or cardiovascular disease.

Baseline characteristics of the individuals undergoing MDCT scanning are presented in Tables 1 and 2. Among the 580 participants, 8.1% had CKD stage 1; 15.7% stage 2; 51.4% stage 3; 19.6% stage 4 and 5.2% stage 5 ND. The median age was 61 [IQR 48–70] years and 39.8% were women. Type 2 diabetes (T2DM) was present in 17.8% and cardiovascular disease in 17.1% of participants, 87.6% had hypertension and 83.3% had hypercholesterolemia, 18.8% were current smokers, 40% were former smokers. Causes of CKD: Chronic glomerulonephritis/vasculitis 28.4%, vascular chronic nephropathy 3.8%, chronic nephropathy in diabetes 10%, chronic tubulointerstitial nephropathy 1.6%, adult polycystic kidney disease 11.6%, other 16%, unknown 28.6%.

**Table 1:** Baseline demographic and clinical characteristics of the participants in the CPH CKD Cohort undergoing MDCT

Variable	All participants	CKD stage 1	CKD stage 2	CKD stage 3	CKD stage 4	CKD stage 5 ND	*p*-values
No. of participants (n, %)	580	47 (8.1)	91 (15.7)	298 (51.4)	114 (19.6)	30 (5.2)	–
Age (y)	61 (48–70)	42 (35–51)	51 (41–63)	63 (53–70)	65 (55–71)	65 (50–70)	< 0.001
Female sex (n, %)	231 (39.8)	22 (46.8)	40 (44)	113 (37.9)	44 (38.6)	12 (40)	0.71
BMI (kg/m2)	28.4 (0.2)	26.5 (0.8)	27.4 (0.5)	28.6 (0.3)	28.7 (0.6)	30.6 (1.3)	0.007
Abdominal circumference (cm)	101.2 (0.6)	92.8 (2.3)	99.1 (1.5)	102.2 (0.9)	102.3 (1.5)	106.6 (2.7)	< 0.001
Systolic BP (mmHg)	133 (1)	130 (3)	127 (1)	134 (1)	131 (2)	146 (3)	< 0.001
Diastolic BP (mmHg)	81 (1)	84 (2)	82 (1)	81 (1)	78 (1)	82 (2)	0.026
Type 1 diabetes (n, %)	11 (1.9)	0 (0)	0 (0)	8 (2.7)	2 (1.8)	1 (3.3)	< 0.001
Type 2 diabetes (n, %)	103 (17.8)	1 (2)	3 (3)	60 (20)	30 (26)	9 (30)	
Hypertension (n, %)	508 (87.6)	35 (74.5)	73 (80.2)	269 (90.3)	103 (90.4)	28 (93.3)	0.004
Hypercholesterolemia (n, %)	483 (83.3)	29 (61.7)	78 (85.7)	259 (86.9)	90 (78.9)	27 (90)	< 0.001
Cardiovascular disease (n, %)	99 (17.1)	4 (8.5)	6 (6.6)	58 (19.5)	22 (19.3)	9 (30)	0.006
Alcohol intake (units/week)	2 (0–8)	2 (0–7)	2 (0–10)	2 (0–9)	1 (0–7)	0 (0–3)	0.035
Never smoker (n, %)	239 (41.2)	23 (48.9)	39 (42.9)	121 (40.6)	46 (40.4)	10 (33.3)	0.81
Former smoker (n, %)	232 (40)	14 (29.8)	35 (38.5)	121 (40.6)	46 (40.4)	16 (53.3)	
Current smoker (n, %)	109 (18.8)	10 (21.3)	17 (18.7)	56 (18.8)	22 (19.3)	4 (13.3)	
Smoking (pack/year)	5 (0–25)	0 (0–15)	2 (0–14)	5 (0–30)	9 (0–31)	10 (0–30)	0.37
Physical activity (n, %)							
1) Inactive	84 (14.5)	3 (6)	6 (6)	38 (13)	26 (23)	11 (37)	< 0.001
2) Low	137 (23.6)	5 (11)	20 (22)	70 (24)	35 (31)	7 (23)	
3) Moderate	292 (50.3)	30 (64)	46 (51)	156 (52)	49 (43)	11 (37)	
4) Vigorous	67 (11.6)	9 (19)	19 (21)	34 (11)	4 (3)	1 (3)	
Medicine (n, %)							
Lipid lowering	251 (43)	8 (17)	28 (31)	147 (49)	50 (44)	18 (60)	< 0.001
Insulin	55 (9.5)	0 (0)	0 (0)	33 (11)	17 (15)	5 (17)	< 0.001
Oral antidiabetic	56 (9.7)	1 (2)	3 (3)	32 (11)	16 (14)	4 (13)	0.03
Antihypertensive	468 (80.7)	29 (62)	68 (75)	249 (84)	96 (84)	26 (87)	0.008
Warfarin	38 (6.6)	2 (4.3)	5 (5.5)	16 (5.4)	14 (12.3)	1 (3.3)	0.10

Values for categorical variables are presented as number (percentages); values for continuous variables are given as mean (SEM) or median (IQR). *P*-values are given by the χ^2^-test, one-way ANOVA or the Kruskal-Wallis test for comparison of CKD stages 1–5. *MDCT* multidetector computed tomography, *BMI* Body Mass index

**Table 2:** Laboratory characteristics of the participants in the CPH CKD Cohort undergoing MDCT

Variable	All participants	CKD stage 1	CKD stage 2	CKD stage 3	CKD stage 4	CKD stage 5 ND	*p*-values
No. of participants (n, %)	580	47 (8.1)	91 (15.7)	298 (51.4)	114 (19.7)	30 (5.2)	–
Creatinine clearance (mL/min)	67 (45–100)	126 (105–147)	118 (96–130)	68 (52–83)	36 (29–46)	22 (18–26)	< 0.001
Urine albumin/creatinine ratio (10^−3^)	105 (19–670)	48 (12–223)	54 (9–543)	73 (13–421)	212 (35–874)	1050 (326–2141)	< 0.001
24-h urine protein (g/d)	0.3 (0.1–1.1)	0.2 (0.1–0.7)	0.2 (0.1–1.0)	0.2 (0.1–0.9)	0.4 (0.2–1.4)	1.8 (0.6–3.5)	< 0.001
P-HDL-C (mmol/l)	1.5 (0.0)	1.7 (0.1)	1.7 (0.1)	1.5 (0.0)	1.4 (0.1)	1.2 (0.1)	< 0.001
P-LDL-C (mmol/l)	3.1 (0.0)	3.2 (0.1)	3.4 (0.1)	3.0 (0.1)	2.9 (0.1)	2.8 (0.2)	0.004
P-triglycerides (mmol/l)	1.5 (1.0–2.2)	1.0 (0.8–2.0)	1.2 (0.9–2.0)	1.6 (1.1–2.2)	1.6 (1.2–2.2)	1.8 (1.4–2.6)	0.008
P-glucose (mmol/l)	5.7 (5.1–6.4)	5.4 (4.8–6.0)	5.5 (5.0–6.0)	5.7 (5.2–6.8)	5.8 (5.2–6.7)	5.8 (5.3–6.9)	< 0.001
P-Ca2+ (mmol/l)	1.22 (0.00)	1.23 (0.01)	1.23 (0.00)	1.22 (0.00)	1.20 (0.01)	1.18 (0.01)	< 0.001
P-phosphate (mmol/l)	1.08 (0.01)	0.98 (0.03)	0.98 (0.02)	1.02 (0.01)	1.20 (0.02)	1.55 (0.05)	< 0.001
P-Mg (mmol/l)	0.84 (0.00)	0.78 (0.01)	0.8 (0.01)	0.83 (0.01)	0.87 (0.01)	0.91 (0.03)	< 0.001
P-PTH (pmol/l)	7.2 (4.7–11.5)	4.4 (3.5–5.4)	4.8 (4.0–6.4)	7.2 (5.1–10.0)	12.7 (8.3–18.9)	25.0 (14.3–30.7)	< 0.001

Values for categorical variables are presented as number (percentages); values for continuous variables are given as mean (SEM) or median (IQR). *P*-values are given by one-way ANOVA or the Kruskal-Wallis test for comparison of CKD stages 1–5. *MDCT* multidetector computed tomography, *P* plasma, *HDL-C* high-density lipoprotein cholesterol, *LDL-C* low-density lipoprotein cholesterol, *Ca2+* ionized calcium, *Mg* magnesium, *PTH* parathyroid hormone

### Regional distribution and severity of arterial calcification

Median calcium scores in all five arterial regions were higher in individuals with CKD stages 3–5 ND as compared with stages 1–2 (*p* < 0.001) (Table 3). The calcium scores were higher in both the thoracic and abdominal aorta, and in the iliac arteries compared with the coronary and carotid arteries.

**Table 3:** Baseline calcium scores in five major arterial sites according to CKD stage

Arterial site	CKD stage 1 *n* = 47	CKD stage 2 *n* = 91	CKD stage 3 *n* = 298	CKD stage 4 *n* = 114	CKD stage 5 ND *n* = 30	*p*-values
Carotid arteries	0 (0–0)	0 (0–5)	24 (0–236)	22 (0–388)	121 (0–618)	< 0.001
Coronary arteries	0 (0–36)	0 (0–37)	35 (0–351)	33 (0–541)	201 (0–1010)	< 0.001
Thoracic aorta	0 (0–16)	0 (0–123)	345 (1–2258)	426 (20–2193)	917 (3–3162)	< 0.001
Abdominal aorta	0 (0–154)	6 (0–942)	603 (9–3449)	776 (73–4162)	861 (26–4289)	< 0.001
Iliac arteries	0 (0–477)	4 (0–1395)	651 (7–2598)	575 (31–5045)	1162 (49–4448)	< 0.001

Values are given as median (IQR). *P*-values are from the Kruskal-Wallis test

**Fig. 1:**
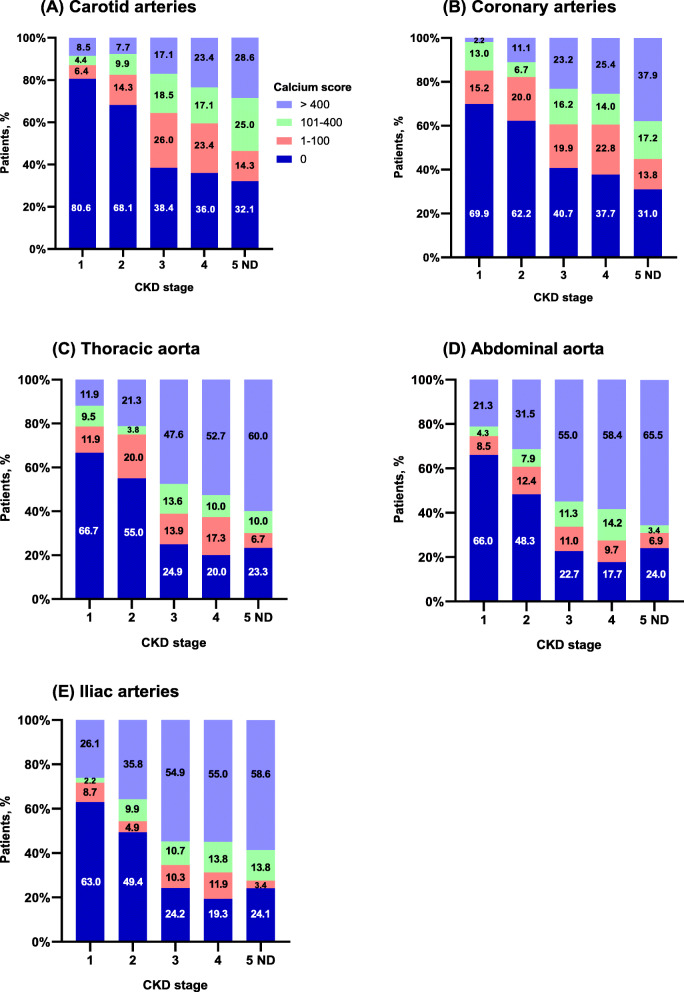
**a**–**e**. Prevalence and severity of calcification in five major arterial sites according to CKD stage. Based on the distribution of Agatston scores the CPH CKD Cohort participants were divided into calcium score categories of 0, 1–100, 101–400 and > 400. **a** Carotid arteries, *n* = 569; **b** Coronary arteries, *n* = 576; **c** Thoracic aorta, *n* = 535; **d** Abdominal aorta, n = 569; **e** Iliac arteries, *n* = 546

Prevalence and severity of calcification in all arterial regions by CKD stage are shown in Fig. 1 a–e and Additional file 1. In all five CKD stages, calcification was most prevalent in both the thoracic and abdominal aorta, and in the iliac arteries. Participants with CKD stages 3–5 ND had the highest prevalence of calcification and the highest frequency of calcium scores > 400 in all arterial sites. Calcification in at least one arterial site was present in 52.4, 70.4 and > 90% of patients with CKD stages 1, 2 and 3, respectively.

Severity and prevalence of calcification was comparably greater in men in all arterial regions (Additional file 2).

### Risk factors of arterial calcification

Participants with calcium score > 400 in all arterial regions were significantly older, had a reduced creatinine clearance and more were men (data not shown). Also, they had a higher prevalence of T2DM, cardiovascular disease, abdominal obesity, reduced physical activity, use of cholesterol-lowering medicine and a greater pack-year history of smoking, as compared with patients with calcium scores ≤400.

We assessed eight cardiovascular risk factors and their association with the relative change in arterial calcification (Table 4 (adjusted data) and Additional file 3 (unadjusted data)). In multivariable models, increasing age was significantly associated with more calcification throughout all arterial sites (*p* for all < 0.001). Smoking and hypertension were independent risk factors of calcification in all arterial regions except for the coronary arteries (*p*-values range between < 0.001 and *p* = 0.01). The same applied for cholesterol-lowering medication (*p*-values range between < 0.001 and *p* = 0.02), except for the carotid arteries (*p* = 0.058). Male sex was only associated with calcification of the coronary arteries (*p* = 0.002) and the iliac arteries (*p* < 0.001), whereas the association between diabetes and calcification was insignificant in all sites. Use of warfarin was associated with calcification of the coronary arteries (*p* = 0.01) only.

**Table 4:** Adjusted associations between cardiovascular risk factors and arterial calcification in the five arterial regions

Variable	Total carotid arteries (*n* = 526)		Total coronary arteries (*n* = 535)		Total thoracic aorta (*n* = 494)		Abdominal aorta (*n* = 525)		Total iliac arteries (*n* = 502)	
	IRR (95%CI)	***P***-value	IRR (95%CI)	***P-***value	IRR (95%CI)	***P***-value	IRR (95%CI)	***P***-value	IRR (95%CI)	***P***-value
Age (y)	1.09 (1.06–1.11)	*0.000*	1.10 (1.07–1.13)	*0.000*	1.20 (1.18–1.23)	*0.000*	1.14 (1.12–1.16)	*0.000*	1.13 (1.10–1.15)	*0.000*
Male sex	0.98 (0.55–1.80)	*0.994*	2.33 (1.35–4.00)	*0.002*	0.87 (0.60–1.27)	*0.482*	1.21 (0.82–1.78)	*0.331*	2.31 (1.55–3.45)	*0.000*
Hypertension	3.17 (1.53–6.55)	*0.002*	1.92 (0.97–3.80)	*0.061*	3.11 (1.73–5.56)	*0.000*	2.67 (1.54–4.62)	*0.000*	2.09 (1.19–3.69)	*0.011*
Diabetes	1.99 (1.01–3.91)	*0.05*	1.68 (0.88–3.22)	*0.119*	0.89 (0.52–1.49)	*0.647*	1.11 (0.66–1.88)	*0.395*	1.29 (0.75–2.21)	*0.353*
Cholestesterol- lowering medication	1.66 (0.98–2.82)	*0.058*	1.88 (1.11–3.20)	*0.020*	2.54 (1.67–3.86)	*0.000*	1.65 (1.09–2.51)	*0.018*	1.66 (1.09–2.55)	*0.019*
Per 10 units increase in pack-years of smoking	1.23 (1.09–1.39)	*0.001*	1.08 (0.97–1.21)	*0.358*	1.29 (1.16–1.43)	*0.000*	1.21 (1.10–1.34)	*0.000*	1.30 (1.17–1.45)	*0.000*
Anticoagulants (Warfarin)	1.36 (0.53–3.48)	*0.517*	3.36 (1.34–8.44)	*0.010*	1.70 (0.82–3.55)	*0.156*	1.65 (0.80–3.42)	*0.117*	1.76 (0.82–3.76)	*0.146*
Per 10 units decrease in creatinine clearance (ml/min)	1.03 (0.96–1.10)	*0.367*	1.08 (1.02–1.14)	*0.012*	1.04 (1.01–1.08)	*0.037*	1.03 (0.98–1.08)	*0.186*	1.01 (0.95–1.06)	*0.797*

*IRR* incidence rate ratio, *CI* Confidence interval

### Declining kidney function and calcium score

**Fig. 2:**
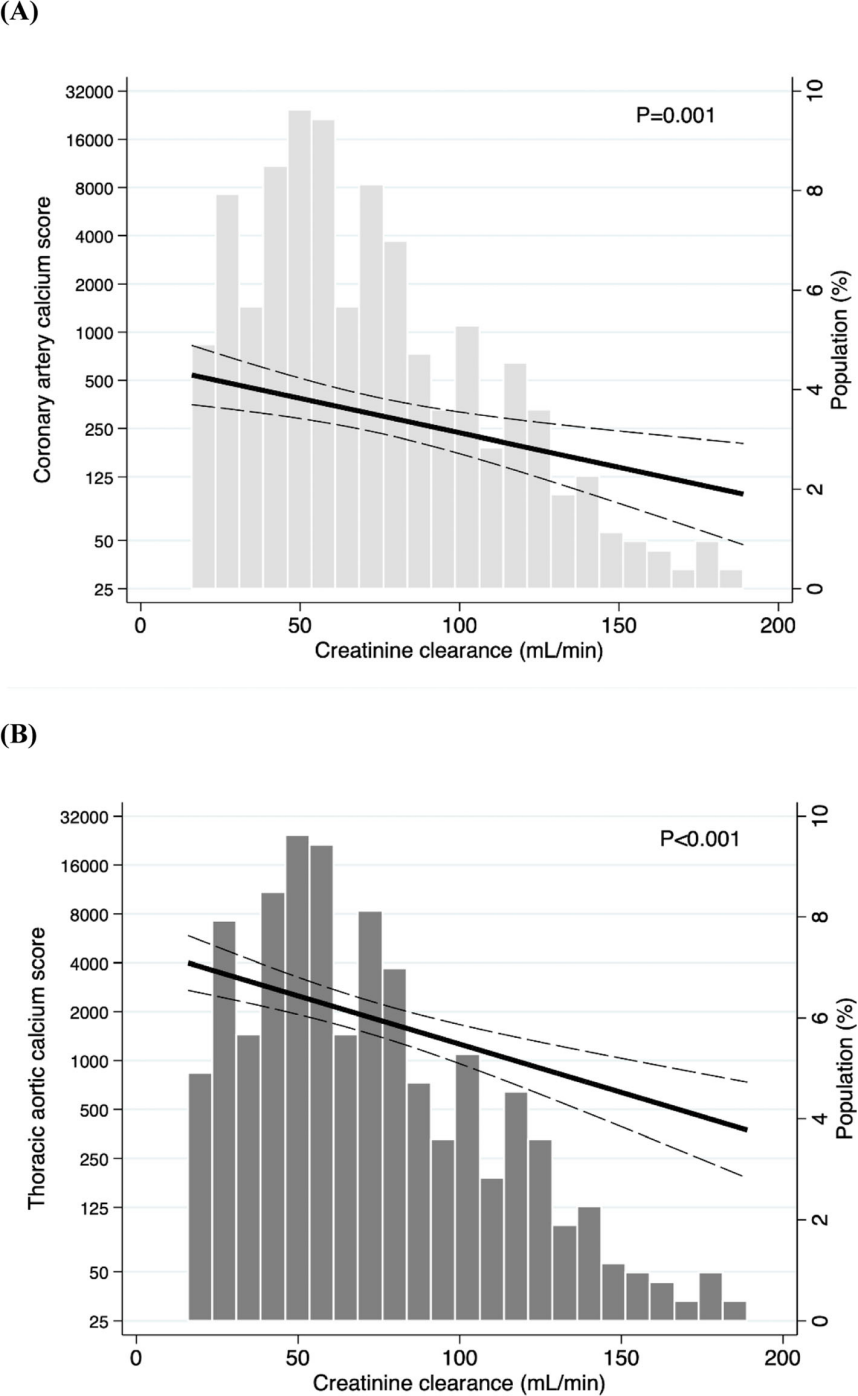
**a**–**b**. Association between kidney function and arterial calcification of the coronary arteries and the thoracic aorta. Negative binomial regression models assessing the association between creatinine clearance (mL/min) and arterial calcium score as a continuous variable in the coronary arteries (**a**) and the thoracic aorta (**b**). Black and dotted lines indicate association correlate and 95% confidence intervals. Histogram indicates the distribution of creatinine clearance (mL/min) in the CPH CKD Cohort

In multivariable models an association between declining creatinine clearance (mL/min) and increasing calcium score was observed in the coronary arteries and the thoracic aorta (Table 4). Per 10 units decrease in creatinine clearance the relative increase in calcium score was 8% in the coronary arteries (95% CI: + 2% to + 14%, *p* = 0.012) and 4% in the thoracic aorta (95% CI: + 1% to + 8%, *p* = 0.037). Figure 2 illustrates this association using the unadjusted results. The same trend in association was shown in the remaining three arterial sites (carotid arteries, abdominal aorta, iliac arteries), however, this pattern was not statistically significant (figures not shown).

In multivariable models no association was found between urine protein excretion and calcification in any of the arterial sites (*p* > 0.05 for all), whereas plasma phosphate was significantly associated with higher calcium score in the iliac arteries only (*p* = 0.04).

### Association with cardiovascular disease

The relation between severity of calcification in a given arterial site and prevalent cardiovascular disease after adjusting for cardiovascular risk factors is shown in Table 5. Calcium scores > 400 in any arterial region were strongly associated with the presence of cardiovascular disease (*p*-values range between < 0.001 and *p* = 0.02). Calcium scores of 101–400 were associated with cardiovascular disease only in the carotid and coronary arteries (*p* = 0.007 and *p* = 0.005, respectively) and the iliac arteries (*p* = 0.04).

**Table 5:** Multivariable logistic regression analysis assessing the association between calcium score and cardiovascular disease

Variable	OR (95% CI)	*p*-value
Carotid arteries		
Calcium score 0	*Reference*	
Calcium score 1–100	2.0 (0.8–4.8)	0.13
Calcium score 101–400	3.4 (1.4–8.3)	0.007
Calcium score > 400	6.0 (2.5–15.1)	< 0.001
Coronary arteries		
Calcium score 0	*Reference*	
Calcium score 1–100	1.1 (0.4–2.8)	0.88
Calcium score 101–400	3.6 (1.5–8.9)	0.005
Calcium score > 400	6.7 (2.9–16.4)	< 0.001
Thoracic aorta		
Calcium score 0	*Reference*	
Calcium score 1–100	1.6 (0.4–6.1)	0.49
Calcium score 101–400	1.3 (0.3–5.4)	0.76
Calcium score > 400	4.4 (1.4–15.9)	0.02
Abdominal aorta		
Calcium score 0	*Reference*	
Calcium score 1–100	1.4 (0.3–6.4)	0.69
Calcium score 101–400	2.5 (0.6–11.3)	0.21
Calcium score > 400	5.0 (1.6–20.0)	0.01
Iliac arteries		
Calcium score 0	*Reference*	
Calcium score 1–100	1.1 (0.1–7.4)	0.90
Calcium score 101–400	5.1 (1.2–28.2)	0.04
Calcium score > 400	8.3 (2.3–41.6)	0.004

Adjusted for age, sex, hypertension, diabetes, hypercholesterolemia, smoking, creatinine clearance (mL/min)

*OR* odds ratio, *CI* Confidence interval

## Discussion

The CPH CKD Cohort Study is the first to examine the distribution and severity of arterial calcification as assessed by CT scanning of multiple arterial sites in individuals with CKD stages 1–5 ND. We demonstrated that calcification in at least one arterial site was present in > 90% of patients with CKD stage 3. In all five CKD stages, calcification was most prevalent in both the thoracic and abdominal aorta, and in the iliac arteries. These arterial sites also showed the highest calcium scores. Arterial calcification in all five arterial sites was more severe in CKD stages 3–5 ND as compared with CKD stages 1–2.

High calcium scores (> 400) in all arterial regions were independently associated with prevalent cardiovascular disease, whereas intermediate calcium scores (101–400) showed the same association only in the carotid, coronary and iliac arteries.

A number of studies have found that CACS improves prediction of cardiovascular events in CKD and provides additional value beyond existing clinical risk factor scoring [8–11]. Moreover, a small study indicated that the addition of coronary and aortic calcification scores improved cardiovascular risk assessment in a CKD population [23].

Follow-up of the CPH CKD Cohort is essential to determine whether calcium scores from extracardiac arteries are better predictors of future cardiovascular events than CACS in individuals with CKD. Possibly, calcium scores from different arterial sites may predict different cardiovascular events, e.g. occlusive arterial disease due to atherosclerosis (mainly intimal calcification) or heart failure due to arterial stiffness (mainly medial calcification). Accordingly, a recent autopsy study revealed that coronary calcium was almost entirely intimal in patients with end stage renal disease [24]. Of note, the calcium score category system applied in the present study has only been validated for CACS [20]. The association of high calcium categories in all arterial sites with prevalent cardiovascular disease indicates that the system potentially could be valuable in extracardial sites, but follow-up studies are encouraged to validate this. Only semiquantitative calcium score category systems have so far been described for all arterial sites, e.g. by Takx et al. [25].

The prevalence of coronary artery calcification in our study agrees well with findings in other studies of patients with CKD [11, 26]. To date, few studies applying CT scans have assessed calcification beyond the coronary arteries. Mizuiri et al. [27] reported calcification in both the coronary (81.4%) and iliac arteries (87.4%) in 145 individuals with a median age of 72 years, a median eGFR of 32 mL/min/1.73 m^2^ and a high prevalence of diabetes and cardiovascular disease. The older age of the population studied by Mizuiri et al. could partly account for their higher prevalence of coronary artery calcification and the smaller difference in prevalence of calcification between the coronary and iliac arteries.

Interestingly, the calcification pattern for patients with advanced CKD in our cohort is in some ways similar to that of men and women aged > 70 years from the general population. Accordingly, Allison et al. [28] assessed arterial calcification by electron beam CT scans in the same five arterial sites in 650 asymptomatic individuals with no renal disease. They found that the prevalence of calcification was highest in the coronary arteries at age < 50 (women) and age < 60 years (men). In men and women aged > 70 years the prevalence of calcification was similar or higher in the aorta as compared to the coronary arteries, and the prevalence of calcification of the iliac arteries was almost as high. Thus, except for a higher prevalence of coronary calcification as compared with other arterial sites in the non-renal population, the similarities between the calcification pattern of the CPH CKD Cohort with a median age of 61 years and that of a population with no renal disease aged > 70 years, suggest accelerated aging of the arterial system in patients with CKD with an early involvement of non-coronary arteries. Likewise, this finding supports the idea that arterial regions other than the coronary arteries should be included when assessing calcification scores in individuals with CKD.

Noteworthy, in our study only age was found to be independent risk factors of arterial calcification throughout all arterial regions. Smoking, hypertension and use of cholesterol-lowering medication were independent risk factors in four out of five arterial sites. The association between arterial calcification and statin use is complex [29]. Warfarin treatment, which has been linked to accelerated medial calcification especially in patients with advanced CKD [30], was an independent risk factor of calcification in the coronary arteries only. This could be due to the small number of patients receiving warfarin in our study (*n* = 38). Plasma phosphate, a well-known risk factor of arterial calcification [31–33], was an independent predictor of arterial calcification in the iliac arteries only. The lack of association with calcification of other arterial sites could be attributable to the well-regulated plasma phosphate levels in our cohort. Further, the study design did not allow us to evaluate the cumulated effect of any long-term fluctuations of plasma phosphate.

We found the association between declining kidney function and increasing calcium score to remain statistically significant only for the coronary arteries and the thoracic aorta upon adjustment for co-variates. The loss of association in the other arterial sites may be a consequence of the strong effect of well-established cardiovascular risk factors on arterial calcification. These risk factors were highly prevalent in the participants with the most advanced CKD stages and the highest calcium scores. Indeed, other studies of the CKD population have shown decreased association between kidney function and CACS after correction for common risk factors [26, 34, 35]. For example, in the CRIC study [26] the association between eGFR and CACS was attenuated by multivariable adjustment but remained statistically significant for eGFR < 30 compared with > 60 ml/min/1.73m^2^.

Likewise, the relatively small sample sizes of participants with CKD stage 1 and 5 ND may attenuate the association between kidney function and calcium scores. Moreover, reported patterns of independent risk factors for calcification differ with arterial sites in both CKD cohorts [27, 36] and the general population [28, 37, 38]. Most likely, the inconsistent patterns reflect differences in the studied populations as well as different methods for registration of risk factors and diverging sensitivity of CT scanning protocols. The true importance of modifiable risk factors can only be determined by intervention studies.

To date, there is no effective therapy for arterial calcification [2]. Strict control of calcium and phosphate metabolism may reduce progression of arterial calcification and cardiovascular disease, but this remains to be convincingly demonstrated [39]. A sensitive method for measuring early arterial calcification in CKD is pivotal for evaluation of biomarkers and interventive measures.

We recognize that the present study had some limitations. First, it is a single centre study with no available follow-up data for the time being, due to the cross-sectional design our results do not provide definite answers related to causation, only association. Second, the relatively small number of participants in CKD stages 1 and 5 may attenuate the association between kidney function and calcium scores. Finally, the CPH CKD Cohort had significantly younger women than men; consequently, this may cause a larger difference between sexes than expected.

The main strengths of this study include its detailed characterisation of participants and that it is the first study exploring the pattern of calcification in five major arterial regions in different stages of CKD. Moreover, the same highly sensitive 320-MDCT scanner was used for examination of all participants.

## Conclusions

In conclusion, this study set out to explore the pattern of arterial calcification in different stages of CKD.

Our results show that arterial calcification is highly prevalent throughout all five CKD stages and is most prominent in both the thoracic and abdominal aorta, and in the iliac arteries. Follow-up of the CPH CKD Cohort is essential to determine whether calcium scores from extracardiac arteries are better predictors of cardiovascular events than CACS in individuals with CKD.

## Supplementary Information

**Additional file 1 Supplementary Table 1**. Prevalence of calcification (defined as calcium score > 0) according to a given arterial site.

**Additional file 2 Supplementary Table 2**. Arterial calcium scores according to sex in five major arterial regions.

**Additional file 3 Supplementary Table 3.** Unadjusted associations between cardiovascular risk factors and arterial calcification in five arterial regions.

## Data Availability

The datasets generated and/or analysed during the current study are not publicly available due to Danish legal restrictions but are available from the corresponding author on reasonable request, provided relevant ethical and legal permissions have been attained priorly and researchers meet the criteria for access to confidential data.

## Abbreviations

CACS: Coronary artery calcium score
CKD: Chronic kidney disease
CPH: Copenhagen
CV: Coefficient of variation
eGFR: Estimated glomerular filtration rate
LDL-C: Low-density lipoprotein cholesterol
MDCT: Multi detector computed tomography
ND: No dialysis
T2DM: Type 2 diabetes
