# Supplementary material for: Regional distribution and severity of arterial calcification in patients with chronic kidney disease stages 1–5: a cross-sectional study of the Copenhagen chronic kidney disease cohort
Source: BMC Nephrol. 2020 Dec 9;21:534. doi: 10.1186/s12882-020-02192-y (PMC7726904; doi:10.1186/s12882-020-02192-y)
Supplement: Supplementary file 1 — Additional file 1 Supplementary Table 1. Prevalence of calcification (defined as calcium score > 0) according to a given arterial site. [file 12882_2020_2192_MOESM1_ESM.docx]

**Additional file 1**

**Supplementary Table 1. Prevalence of calcification (defined as calcium score >0) according to a given arterial site.**

| Arterial site | All patients | CKD stage 1 | CKD stage 2 | | CKD stage 3 | CKD stage 4 | CKD stage 5 ND | *p*-value |
| --- | --- | --- | --- | --- | --- | --- | --- | --- |
| Carotid arteries  (n, %) | 308 (54.1) | 9 (19.1) | 29 (31.7) | 180 (61.6) | | 71 (64) | 19 (67.9) | <0.001 |
| Coronary arteries  (n, %) | 320 (55.6) | 14 (30.4) | 35 (38.9) | 179 (60.3) | | 72 (63.2) | 20 (69) | <0.001 |
| Thoracic aorta  (n, %) | 367 (68.6) | 37 (33.3) | 37 (46.3) | 205 (75.1) | | 88 (80) | 23 (76.7) | <0.001 |
| Abdominal aorta  (n, %) | 402 (69.3) | 16 (34) | 46 (51.7) | 225 (77.3) | | 93 (82) | 22 (75.9) | <0.001 |
| Iliac arteries  (n, %) | 381 (69.8) | 17 (37) | 41 (50.6) | 213 (75.8) | | 88 (80.7) | 22 (75.9) | <0.001 |

*P*-values are from the χ^2^-test for categorical variables.
