# Supplementary material for: Regional distribution and severity of arterial calcification in patients with chronic kidney disease stages 1–5: a cross-sectional study of the Copenhagen chronic kidney disease cohort
Source: BMC Nephrol. 2020 Dec 9;21:534. doi: 10.1186/s12882-020-02192-y (PMC7726904; doi:10.1186/s12882-020-02192-y)
Supplement: Supplementary file 2 — Additional file 2 Supplementary Table 2. Arterial calcium scores according to sex in five major arterial regions. [file 12882_2020_2192_MOESM2_ESM.docx]

**Additional file 2**

**Supplementary Table 2. Arterial calcium scores according to sex in five major arterial regions.**

| Variable | All patients | Men | Women | *p*-values |
| --- | --- | --- | --- | --- |
| No. of participants (n, %) | 580 | 349 (60.2) | 231 (39.8) | - |
| Age (y) | 61 (48-70) | 63 (51-70) | 55 (44-67) | <0.001 |
|  |  |  |  |  |
| Calcium scores |  |  |  |  |
| Carotid arteries | 4 (0-213) | 40 (0-337) | 0 (0-38) | <0.001 |
| Coronary arteries | 12 (0-264) | 63 (0-622) | 0 (0-60) | <0.001 |
| Thoracic aorta | 169 (0-1827) | 293 (0-2399) | 70 (0-1119) | 0.005 |
| Abdominal aorta | 394 (0-2839) | 792 (5-3937) | 112 (0-1496) | <0.001 |
| Iliac arteries | 383 (0-2651) | 968 (22-4038) | 77 (0-824) | <0.001 |

Values for categorical variables are given as number (percentages); values for continuous variables are given as median (IQR). *P*-values are given for Mann-Whitney U test between groups.
