# Supplementary material for: Regional distribution and severity of arterial calcification in patients with chronic kidney disease stages 1–5: a cross-sectional study of the Copenhagen chronic kidney disease cohort
Source: BMC Nephrol. 2020 Dec 9;21:534. doi: 10.1186/s12882-020-02192-y (PMC7726904; doi:10.1186/s12882-020-02192-y)
Supplement: Supplementary file 3 — Additional file 3 Supplementary Table 3. Unadjusted associations between cardiovascular risk factors and arterial calcification in five arterial regions. [file 12882_2020_2192_MOESM3_ESM.docx]

**Additional file 3**

**Supplementary Table 3. Unadjusted associations between cardiovascular risk factors and arterial calcification in the five arterial regions.**

|  | Carotid arteries  (n=526) | | Coronary arteries  (n=535) | | Thoracic aorta  (n=494) | | Abdominal aorta  (n=525) | | | Iliac arteries  (n=502) | |
| --- | --- | --- | --- | --- | --- | --- | --- | --- | --- | --- | --- |
| Variable | IRR (95%CI) | ***p*** | IRR (95%CI) | ***p*** | IRR (95%CI) | ***p*** | | IRR (95%CI) | ***p*** | IRR (95%CI) | ***p*** |
| I. Age, per 1-year increase | 1.11 (1.09-1.13) | <0.001* | 1.12 (1.11-1.15) | <0.001* | 1.23 (1.21-1.26) | <0.001* | | 1.17 (1.14-1.19) | <0.001* | 1.16 (1.13-1.18) | <0.001* |
| II. Male sex | 2.81 (1.71-4.62) | <0.001 | 5.20 (3.20-8.47) | <0.001* | 2.16 (1.36-3.45) | 0.001 | | 2.03 (1.34-3.07) | 0.001 | 3.00 (1.96-4.60) | <0.001* |
| III. Hypertension | 2.16 (1.03-4.56) | 0.042* | 2.14 (1.02-4.49) | 0.045 | 2.98 (1.48-5.98) | 0.002* | | 1.86 (1.01-3.44) | 0.047* | 2.08 (1.10-3.94) | 0.025* |
| IV. Diabetes | 2.32 (1.25-4.28) | 0.007 | 2.59 (1.40-4.80) | 0.002 | 1.93 (1.10-3.42) | 0.023 | | 1.87 (1.12-3.12) | 0.016 | 2.24 (1.32-3.79) | 0.003 |
| V. Cholesterol lowering medication | 2.86 (1.76-4.67) | <0.001 | 3.50 (2.15-5.68) | <0.001* | 4.02 (2.57-6.30) | <0.001* | | 2.84 (1.90-4.26) | <0.001* | 3.20 (2.10-4.87) | <0.001* |
| VI. Smoking, per 10 package year increase | 1.35 (1.19-1.53) | <0.001* | 1.22 (1.08-1.37) | 0.001 | 1.36 (1.21-1.53) | <0.001* | | 1.28 (1.16-1.42) | <0.001* | 1.39 (1.25-1.54) | <0.001* |
| VII. Anticoagulants (Warfarin) | 1.66 (0.61-4.51) | 0.32 | 2.76 (1.02-7.50) | 0.046* | 1.74 (0.69-4.38) | 0.24 | | 1.73 (0.76-3.96) | 0.20 | 1.87 (0.80-4.38) | 0.15 |
| VIII. Creatinine clearance, per 10 units decrease | 1.13 (1.06-1.20) | <0.001 | 1.10 (1.04-1.17) | 0.001* | 1.15 (1.09-1.21) | <0.001* | | 1.14 (1.08-1.20) | <0.001 | 1.11 (1.05-1.17) | <0.001 |
| *Remained significant in multivariable models adjusted for parameters I-VIII. IRR: incidence rate ratio, CI: confidence interval | | | | | | | | | | | |
